# Confining vertical conducting filament for reliable resistive switching by using a Au-probe tip as the top electrode for epitaxial brownmillerite oxide memristive device

**DOI:** 10.1038/s41598-018-37986-6

**Published:** 2019-02-04

**Authors:** Venkata Raveendra Nallagatla, Janghyun Jo, Susant Kumar Acharya, Miyoung Kim, Chang Uk Jung

**Affiliations:** 1 Department of Physics and Oxide Research Center, Hankuk University of Foreign Studies, Yongin, 449-791 Korea; 2 Department of Material Science and Engineering and Research Institute of Advanced Materials, Seoul National University, Seoul, 151-747 Korea

## Abstract

We had discovered novel resistance switching phenomena in SrCoO_*x*_ epitaxial thin films. We have interpreted the results in terms of the topotactic phase transformation between their insulating brownmillerite phase and the conducting perovskite phase and the existence of a rather vertical conducting filament due to its inherent layered structure. However, the rough interface observed between the SrCoO_*x*_ and the Au top electrode (area ~10000 μm^2^) was assumed to result in the observed fluctuation in key switching parameters. In order to verify the effect of rough interface on the switching performance in the SrCoO_*x*_ device, in this work, we studied the resistive switching properties of a SrCoO_*x*_ device by placing a Au-coated tip (end area ~0.5 μm^2^) directly on the film surface as the top electrode. The resulting device displayed much improved endurance and showed high uniformity in key switching parameters as compared to the device having a large top electrode area. A simulation result confirmed that the Au-coated tip provides a local confinement of the electrical field, resulting in confinement of oxygen ion distribution and therefore localization of the conducting filament. By minimizing other free and uncontrollable parameters, the designed experiment here provides the most direct and isolated evidence that the rough interface between electrode and ReRAM matrix is detrimental for the reproducibility of resistivity switching phenomena.

## Introduction

Resistive random access memory (ReRAM) with a simple metal-insulator-metal structure shows promising characteristics in terms of scalability, low power operation, and multilevel data storage capability, and it is suitable for next-generation memory applications^1–3^. Despite significant advances in ReRAM over the last few years, its practical implementations have been hindered due to some fundamental concerns such as the wide distribution of the switching parameters and insufficient endurance (number of switching cycles) as well as its poorly understood mechanism^3,4^. In many ReRAM devices, the essential mechanism is based on the formation and rupture of the conducting filament (CF) formed between two electrodes, resulting in repeatable resistive switching between high-resistance states (HRS) and low-resistance states (LRS)^5–10^. In many oxide ReRAM devices, the formation of the CF is triggered by field-assisted oxygen ion migrations, resulting in a change in the electronic conductivity of the switching device. Nevertheless, based on the current understanding of resistive switching (RS), a better control of oxygen ion or vacancy generation rates and the distribution dynamics is necessary to improve the reproducibility and stability (cycle-to-cycle) of the RS device performance^11,12^.

Many researchers have attempted to understand and control oxygen ion dynamics through various methods^11,13–16^. These methods include the insertion of metal nanodots or nanoparticles^14^, employing a nanovia electrode structure^16^ and geometrically embedding nanotip electrodes^11^. All these methods have been used to control the O^2−^ ions or vacancies generation rate and distribution, so that the CF was confined along the same path as the preferential sites. For example, Ru and Pt nanodots have been embedded in the TiO_2_ layer sandwiched between two Pt electrodes^13,14^ to create preferential sites for localizing the electrical fields, so that the formation of CFs was initiated from the nanodots. Furthermore, in nanodevices like Pt/HfO_2_/TiN, a nanovia electrode structure with a nm-size electrode was employed to generate a geometrical confinement of the CF^16^. Similarly, Gang *et al*. reported an effective geometric approach with a nanotip bottom electrode (BE) to confine the CF with a localized electrical field above the nanotip BE region. Thus, they could achieve a switching device with a highly stable endurance and retention^11^. These approaches suggest that localizing the potential field distribution around the inhomogeneity (doping metal nanoparticle) or nanostructure leads to better control of the oxygen ion generation and distribution^10^. As a consequence, the CF can be confined along the same path at each voltage stress, which causes stable RS behavior in these devices.

The morphology of the surface and interface can alter the operational characteristics of microelectronic devices significantly. For example, it has been reported that a rough surface morphology can increase the leakage current^17^, reduce breakdown voltage^18^, and reduce the yield in thin film oxide capacitance^19^. However, there are conflicting reports on the effect of surface morphology of the top and bottom electrodes on ReRAM performance. For example, it has been reported that increasing the roughness at the metal/oxide interface can decrease the variability in the switching parameters of a ReRAM device including set and reset voltages and HRS and LRS resistances^20^. Similarly, Kim *et al*. showed that using a rougher substrate is effective in reducing the operating voltage current^21^. In contrast, Sanjoy *et al*. demonstrated that increasing the roughness of the BE leads to an increase in the failure rate of the device, and consequently, poor switching reliability in the performance of the device^18^. They also observed a wider distribution of switching parameters for the rougher electrode.

On the other hand, it has been suspected that the large variation of switching parameters in the memristive device is a result from thermodynamical instability of the filament and especially generation subfilamentary network with different size and shape for successive swiching cycles^22^. Recently, Lee *et al*. and Cho *et al*. showed the preferential generation and confinement of the CF can be realized by engineering the oxygen vacancies along the SrTiO_3_-Sm_2_O_3_ vertical interface, consequently, a better control over the device switching performance with high uniformity and reproducibility^23,24^. Therefore, if the nanoscale conducting channels confined within well-defined path and shape, generation/recombination of oxygen vacancies within the CF can be identical for each switching operation. Then, improved uniformity and better reproducibility in switching can be obtained.

Recently, we observed the bipolar RS behavior of brownmillerite SrCoO_*x*_ (BM-SCO) thin film and found that the forming voltage (*V*_*F*_) could be lower than the set voltage (*V*_*S*_)^25^. This, along with diverse temperature-dependent resistivity behaviors in the LRS and HRS, led us to a new type of switching mechanism based on a local topotactic phase transformation of the SCO from a brownmillerite to perovskite structure. However, the device with BM-SCO thin film exhibited poor repeatability (cycle-to-cycle) and non-uniform switching characteristics, while the conducting films seem to have vertical shape due to its layered structure. It is worth pointing out here that the BM-SCO thin film does not grow atomically flat despite the fact that the underlying SrTiO_3_ (STO) (001) substrate and SrRuO_3_ (SRO) BE are atomically flat; therefore, the Au/BM-SCO interface roughness can produce many local preferential sites due to concentered electrical fields in the vicinity of randomly distributed asperities under the whole top electrode (TE) region. As a result, the randomly generated CFs with unconfined oxygen ion distributions lead to poor switching performance in the BM-SCO device with a large TE area. On the other hand, we also observed the RS behavior in *Au*(100 μm)/SrFeO_*x*_/SRO in which SrFeO_*x*_ thin film was grown with an atomically smooth surface^26^. This SrFeO_*x*_ thin film device exhibited better reproducible switching behavior with narrow distribution of the switching characteristics even in a large TE area (100 μm × 100 μm), due to the reduced probability of random generation of CFs.

**Figure 1:**
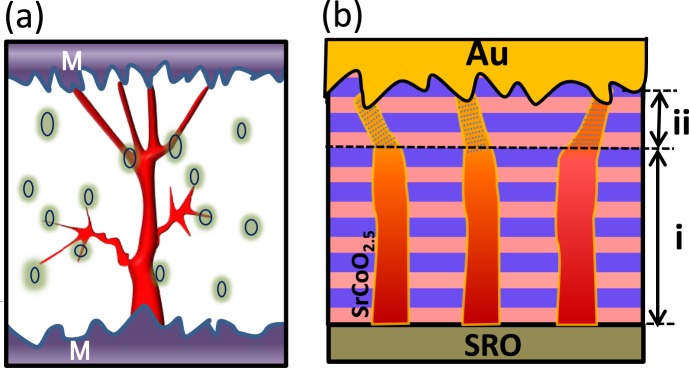
Schematics of conducting filament formation in (**a**) polycrystalline thin films, and (**b**) brownmillerite epitaxial SrCoO_x_ thin film, where, a region (i) with identical and vertical of CFs and (ii) non-uniform CFs.

In devices consisting of polycrystalline thin films, there exist many more degrees of freedom on the creation and rupture of CF. The randomness comes from three part; rough interface adjacent to TE, rough interface adjacent to BE, and the ReRAM layer having random and local defects. These three sources of freedom work together to result in random and uncontrollable CF as shown in Fig. 1(a). So separation and understanding of each contribution is almost impossible. During ReRAM switching, the BM-SCO layer was suggested to have rather vertical CF due to its inherent layered structure, benefit of quantum confinement effect, and atomically flat interface adjacent to BE, shown in Fig. 1(b) (even though the dendrite-like structure may form near the top surface region (ii) due to the presence of many preferential local passes). Here benefit of quantum confinement means that by changing a tetrahedral layer inside BM-SCO, you can get three connected octahedral layers and thus much more conducting properties. [In the well-established critical thickness debate on ultrathin SrRuO_3_ film, it is well known that the resistance of 3 unit cell think SRO is well not just 3 times smaller than 1 unit cell thick SrRuO3 layer.] It should be noted that BM-SCO should be a good model system for studying the roughness effect on RS to polycrystalline thin films due to following two reasons: (1) For our single crystalline epitaxial BM-SCO thin films, the defects are minimized and the bottom interface of our device is atomically smooth (due to the atomically smooth growth of the SRO BE) and (2) vertical nature of CF due to layered BM-SCO structure. Thus, the device with BM-SCO should provide an ideal platform to study the roughness effect using the Au probe tip.

In this study, the Au-coated probe tip as a mobile TE was placed directly on the top surface of BM-SCO layer to examine the RS by localizing the electrical field under the tip region and minimizing the surface roughness effect on the device performance. The resulting BM-SCO device with placement of the Au-tip exhibited higher retention, and higher repeatable and reliable switching performance. Although this geometry, with the Au tip placed directly on the BM-SCO surface, is different from conventional metal-insulator-metal structures, this approach still provided a possible understanding of RS phenomena and the electrode/interface roughness effect on device performance. To estimate the real contact area of the tip, we performed AFM of surface morphology of BM-SCO active layer before and after loading the tip. The Tip made scratch with ~2.5 nm maximum depth. The scratch was found to consist of a long shallow narrow tail (width ≪1 µm, length ~2 µm) and a circular dent of ≤0.5 µm diameter.

## Results and Discussion

**Figure 2:**
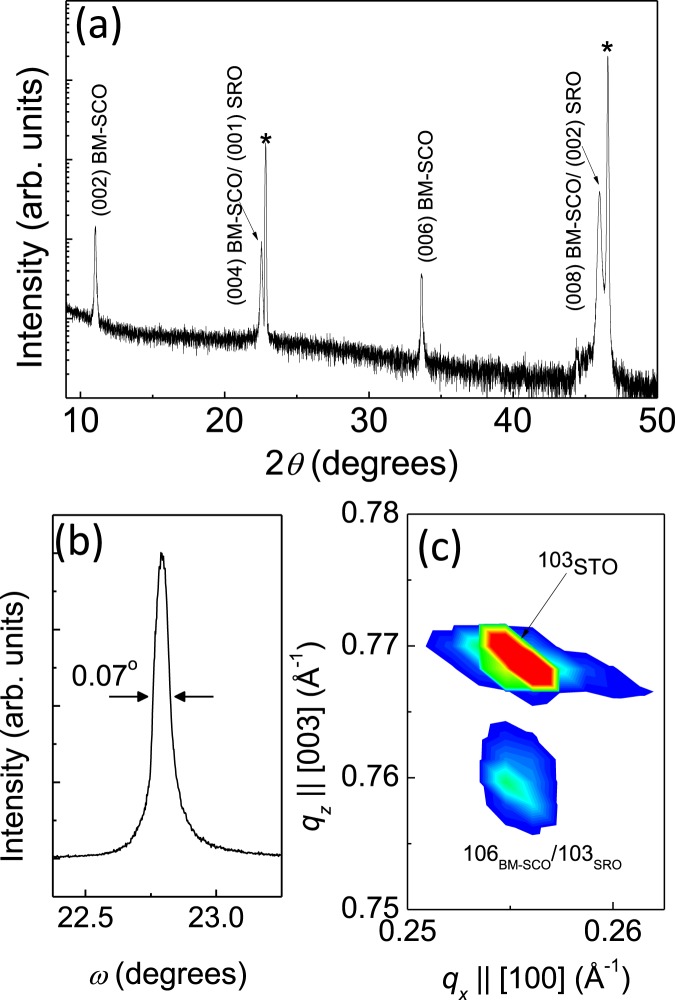
(**a**) X-ray diffraction (XRD) *θ*–2*θ* patterns of as-deposited thin film of the BM-SCO/SRO/STO (001) substrate. The asterisks indicate peaks in the STO substrate; (**b**) XRD rocking curves of the (008) diffraction peak of the brownmillerite SrCoO_2.5_ film; (**c**) reciprocal space maps near the (103) STO Bragg reflection.

Figure 2(a) shows X-ray diffraction (XRD) *θ-2θ* scan patterns for the BM-SCO/SRO/STO (001) substrate heteroepitaxial thin film. The diffraction peak of the (008) plane of the BM-SCO nearly overlapped with the peak corresponding to the (002) planes of the SRO. In addition to the (004) and (008) peaks observed in the XRD pattern of the BM-SCO thin film, we also observed that 2-fold superstructure peaks such as (002) and (006) were caused by the ordering of oxygen defects (these peaks are the unique “fingerprint” indicating the presence of the brownmillerite phase), which suggests that the thin film is a *c*-axis-oriented BM-SCO_._ The calculated average out-of-plane lattice constant was 3.951 × 4 Å, from the (002), (004), (006), and (008) BM-SCO diffraction peaks. No other Bragg diffraction peaks were observed, suggesting no secondary phase exists in a heteroepitaxial oxide structure. Note that bulk BM-SCO is orthorhombic with lattice constants of *a*_o_ = 5.5739 Å, *b*_o_ = 5.4697 Å, and *c*_o_ = 15.745 Å^27^, which can also be represented as pseudo-tetragonal (*a*_t_ = 3.9047 Å and *c*_t_/4 = 3.9363 Å). It is very clear that the BM-SCO thin film could be preferentially grown on an STO (001) substrate (*a* = 3.905 Å) with the *c*_o_-axis along the surface normal direction due to the nearly zero in-plane mismatch. This was clearly seen in XRD patterns as shown in Fig. 2(a).

The XRD rocking curve of the (008) BM-SCO peak as shown in Fig. 2(b), revealed a full width at half maximum of =0.07 degrees, which demonstrated that the film had very high out-of-plane crystallinity. To characterize the epitaxial feature, the reciprocal space mapping around the STO (103) plane reflection was carried out for the BM-SCO/SRO/STO (001) heterostructure as shown in Fig. 2(c). The lower region in the mapping shows thin film peaks and upper peaks corresponding to the strong STO substrate peak. The mapping results show that the BM-SCO/SRO bilayer has grown coherently as its in-plane lattice constant values are the same as those of the underlying STO (001) substrate. Because the lattice constants of both BM-SCO and SRO are slightly larger than that of STO, the as-grown films were under relatively smaller compressive strain. All these results are quite consistent with our previous XRD results on BM-SCO^25^.

**Figure 3:**
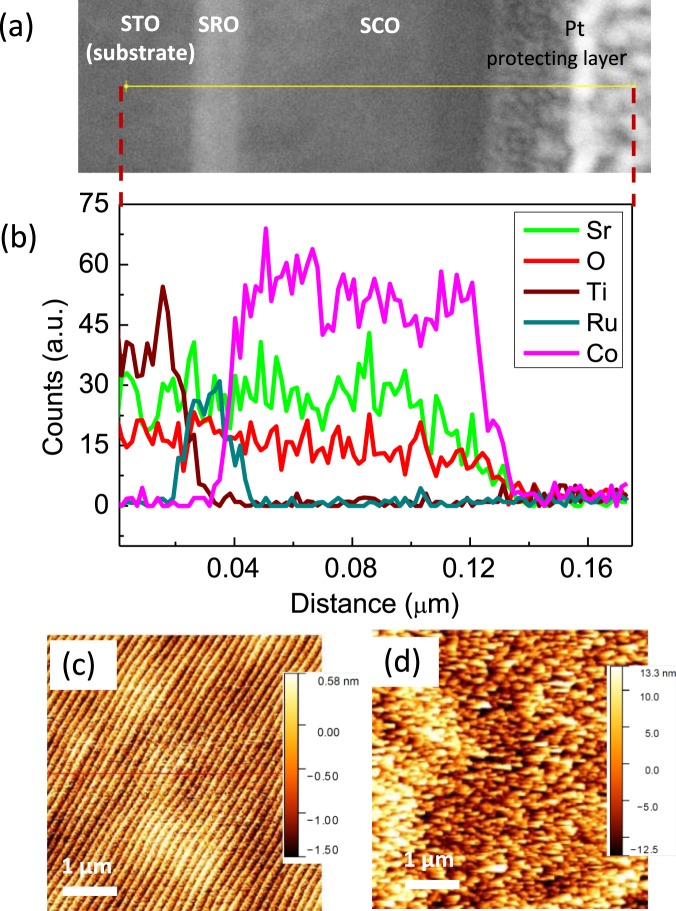
(**a**) Cross-sectional TEM images of the SrCoO_*x*_/SrRuO_3_/SrTiO_3_ (001) heterostructure thin film and (**b**) the corresponding EDS spectra. (**c**) Surface morphology of the treated STO (001) substrate with a clear step terrace. (d) AFM image of the as-grown SrCoO_*x*_ thin film surface. The RMS roughness was ~2 nm.

Figure 3(a) shows a cross-sectional TEM image of BM-SCO/SRO films grown on a STO (001) substrate. The TEM image exhibited atomically flat interfaces, and sharp boundaries between the SRO and STO layers and between the BM-SCO and SRO layers. The observed thicknesses of the SRO and BM-SCO layers were 30 nm and 100 nm, respectively. The concentration profile of the atomic elements was measured by EDS along the yellow line in Fig. 3(a), as shown in Fig. 3(b). It can be seen that the EDS signals of Ti (wine line), Ru (dark cyan line), and Co (magenta line) match well with the corresponding sharp boundaries of the BM-SCO/SRO/STO (001) layers, indicating that there is no appreciable inter-diffusion of atoms. While the Sr (green line) EDS signal was mostly constant across the BM-SCO/SRO/STO film, the O (red line) slightly decreases in the BM-SCO layer region compared to the SRO/STO layer; this means that BM-SCO is in an oxygen deficient phase compared to SRO and STO. The surface morphology of the STO (001) substrate and BM-SCO film was investigated by AFM as shown in Fig. 3(c,d), respectively. A clear step and terrace morphology was observed for the STO (001) substrate, including a 0.4 nm step height with sharp edges. In contrast to the surface of the STO (001) substrate, the surface of the BM-SCO film exhibited a rather rough surface, as shown in Fig. 3(d). The root-mean-square roughness was ~2 nm, and we observed large fluctuations (3–6 nm) in the height profile of the BM-SCO film surface (data not shown). Here, we note that the BM-SCO unfortunately does not have layer by layer growth behavior which is know from the first report^27^.

**Figure 4:**
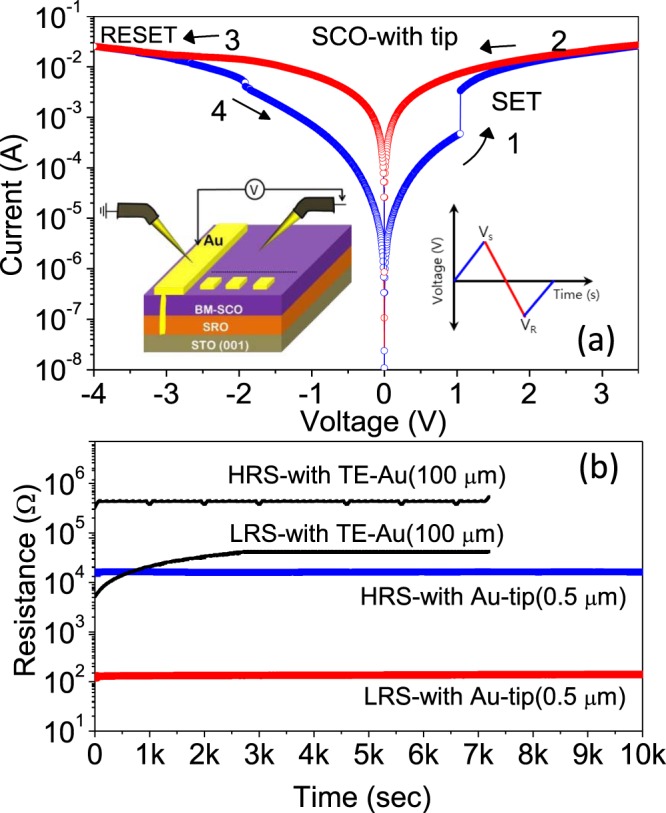
(**a**) Current–voltage switching curves for the SrCoO_*x*_/SrRuO_3_ structure using a Au-coated tip, measured with a compliance current of 100 mA. The arrows indicate the directions of the voltage sweeps. The inset shows the schematic for our SrCoO_*x*_/SrRuO_3_ structure with the Au-plated probe tip placed directly on the SrCoO_*x*_ surface. (**b**) Retention data for the low-resistance state (LRS) and the high-resistance state (HRS) for the *Au-tip*(0.5 μm)/BM-SCO/SRO device and *Au*(100 μm)/BM-SCO/SRO.

Representative *I-V* characteristics of bipolar switching in our BM-SCO thin film are presented in Fig. 4(a). A two terminal method with a Au tip as the TE was used for the *I-V* measurement at room temperature, which is shown in the inset of Fig. 4(a). (The diameter of the tip end was estimated to be in the order of ~0.5 μm. We call this configuration a *Au-tip* (0.5 μm)/BM-SCO/SRO device.) The forward bias is defined by the current flow from the TE to the BE, and the negative bias is defined as the opposite. The Au-coated tip was placed directly on the BM-SCO surface, which acts as a mobile TE and the SRO as the BE. Note the SRO BE connected to the wide Au pad, which was grounded throughout the measurements. Before initiating resistive switching (RS) in the BM-SCO/SRO structure, an electrical forming process is necessary to activate the pristine device into the LRS, which was achieved by sweeping the positive voltage ranging from ~1.0 to 2.0 V; this was discussed in detail our previous articles^25,26^. After an initial forming process, when the negative bias is further swept back to zero, the device reverts to a HRS at ~−2.5 V, which is called the “reset” process. Then, by sweeping back to positive voltage bias over a value of ~1.1 V, an abrupt increase in current is observed and the device is switched back to the LRS again, which is known as the “set” process, as shown in Fig. 4(a).

We first confirmed the retention capability for the *Au*(100 μm)/BM-SCO/SRO device, as presented by the black lines in Fig. 4(b). (Here, the Au TE area was ~100 μm × 100 μm. The resistances were measured at a reading voltage of 0.2 V.) The HRS/LRS resistance ratio was around 50 initially, and the resistance of the LRS then gradually decayed to around 3 × 10^3^ seconds; the ratio then remained constant at about 10 up to 7 × 10^3^ seconds. Thus, the average HRS/LRS resistance ratio at the saturated status was slightly less than 10. These trends are consistent with our previous report^25^. The decay of the resistance in the LRS state during the initial stage has been explained in other ReRAM devices^28,29^. A large number of weak CFs may be formed in the LRS state due to the larger rough surface area of the interface; however, the unlocalized oxygen ions constituting the CFs may be dispersed away from the weakly generated CFs over time, consequently decaying the resistance of the LRS state.

Using exactly the same sample, but with a Au-coated tip as the TE, we tested the retention of the bare BM-SCO device, which was not covered by a Au electrode. A superior retention capability was observed in our *Au-tip*(0.5 μm)/BM-SCO/SRO device compared to that in the *Au*(100 μm)/BM-SCO/SRO device, as shown in Fig. 4(b). The HRS/LRS resistance ratio for the *Au-tip*(~0.5 μm)/BM-SCO/SRO device was more than 100 and retained its stability without obvious degradation even in the initial stages.

The negligible amount of LRS retention fatigue in the *Au*(~0.5 μm)/BM-SCO/SRO device compared to that in the *Au*(100 μm)/BM-SCO/SRO device could be explained as follows. A strong electric field at the end of the sharp tip may generate a much smaller number (even one or two) of paths for the CFs and these CFs can survive after subsequent switching. The relatively non-localized field in the *Au*(100 μm)/BM-SCO/SRO may generate a greater number of paths for the CFs and not all of them will survive during the subsequent switching; only the best ones will survive, leading to the saturated value of the resistance in the LRS.

The resistances in the LRS and HRS of the *Au-tip*(0.5 μm)/BM-SCO/SRO device are one order of magnitude less compared to those of the *Au*(100 μm)/BM-SCO/SRO device, pointing to the formation of *stronger CF* with high concentration of oxygen ions due to localized filament under the *Au-tip* area. In fact, it has been demonstrated and quantified that the resistance of values of the LRS and HRS are extremely depending on the oxygen ion/vacancy concentrations in the filament region^30^. Whereas, The *Au*(100 μm)/BM-SCO/SRO device with interface roughness promotes many local preferential sites at the *Au*(100 μm)/BM-SCO interface, which leads to poor oxygen ion concentration in CFs region. Consequently, the resistance values of LRS and HRS in the *Au*(100 μm)/BM-SCO/SRO device are one order of magnitude higher as compare to *Au-tip*(0.5 μm)/BM-SCO/SRO device. Additionally, The variation of TE area can also contribute to the difference in resistance, because the resistance in the *Au*(100 μm)/BM-SCO/SRO device consists of two parallel circuits: a matrix and a CF. Thus, the on–off ratio generally becomes higher as the size of the TE decreases.

**Figure 5:**
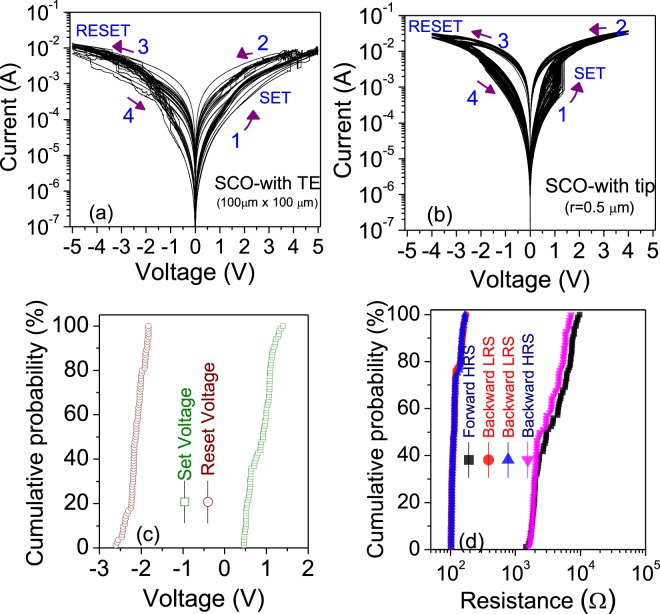
*I-V* curves for the switching cycles in (**a**) the *Au*(100 μm)/BM-SCO/SRO device and (**b**) the *Au-tip*(0.5 μm)/BM-SCO/SRO device. (**c**) Cumulative plots of the set and reset voltages for (**b**); (**d**) Cumulative probability graphs for the HRS and LRS for the forward and reverse biases for (**b**).

To understand the switching reliability and stability in SCO ReRAM, it is important to investigate the statistical distribution of the switching memory parameters such as the resistance values at HRS and LRS (*R*_HRS_ and *R*_LRS_)_,_ *V*_*S*_, and the reset voltages (*V*_*R*_). Figure 5(a,b) depicts typical hysteresis *I-V* loops for 30 consecutive cycles in the *Au* (100 μm)/BM-SCO/SRO device and those for 100 consecutive cycles in the *Au-tip*(0.5 μm)/BM-SCO/SRO, respectively. The switching curve for the *Au*(100 μm)/BM-SCO/SRO device showed poor reproducibility. Moreover, we observed multiple increments and decrements of abrupt current levels during a positive (set) and negative bias (reset) sweep in the device, which might be due to the possibility of multiple weak CFs existing inside the BM-SCO active layer^31^. On the other hand, the *Au-tip*(0.5 μm)/BM-SCO/SRO device showed good RS behavior, and the device could be switched for more than 100 cycles. The results indicated that by reducing the electrode area in our metal-insulator-metal structure using the point contact method, the RS stability and repeatability was drastically increased.

To further elucidate the switching stability of the *Au-tip*(0.5 μm)/BM-SCO/SRO device, we calculated the cumulative probability distribution for *R*_HRS_. *R*_LRS_, *V*_*S*_, and *V*_*R*_, and the results are plotted in Fig. 5(c,d), and correspond to the *I-V* curves for 100 loops, as shown in Fig. 5(b). The cumulative probability distribution of *V*_*S*_ and *V*_*R*_ was quite narrow: the *V*_*S*_ ranges from 0.46 to 1.45 V with mean (*μ*) and standard deviations (*σ*) of 0.86 and 0.21 V, respectively. The *V*_*R*_ ranges from −2.60 V to −1.82 V with μ = −2.12 V and *σ* = 0.15 V. Our *Au-tip*(0.5 μm)/BM-SCO/SRO device exhibited high uniformity in *V*_*R*_ and *V*_*S*_ with small *σ* values, indicating its highly improved stability over the *Au*(100 μm)/BM-SCO/SRO device.

The above trend can also be verified by studying the fluctuations in the *R*_LRS_ and *R*_LRS_ values. Figure 5(d) shows the cumulative probability distribution of the HRS and LRS resistance in our *Au-tip*(0.5 μm)/BM-SCO/SRO device measured at 0.20 V at forward bias and negative bias, respectively. The measured *R*_LRS_ and *R*_LRS_ featured a low relative fluctuation (*σ*/*μ*). The relative fluctuation values are 0.13, 0.13, 0.42, and 0.41 for positive *R*_LRS_, negative *R*_LRS,_ positive *R*_HRS_ and negative *R*_HRS_, respectively.

These results indicates superior stability and repeatability in our *Au-tip*(0.5 μm)/BM-SCO/SRO device compared with the *A*u(100 μm)/BM-SCO/SRO device. This result reconfirms our previous assertion that a strong field at the end of the sharp tip may generate comparatively smaller but stronger CFs and these CFs can survive subsequent repeated switching. The non-localized field in *Au*(100 μm)/BM-SCO/SRO may generate a greater number of CFs, and not all of them survive during subsequent switching; only the best ones survive and give the saturated value for R_*LRS*_ as shown Fig. 4(b). This explanation is based on the assertion that the surface of BM-SCO is rough; thus, there can be many local minimum paths for CFs in the case of the *Au* (100 μm)/BM-SCO/SRO device, especially near the TE. In contrast, the *Au-tip*(0.5 μm)/BM-SCO/SRO device showed highly reproducible and stable RS performance by minimizing the surface roughness effect with a localized potential field and enhanced control over ion generation and distribution around the tip/surface region.

In contrast to BM-SCO device, the SrFeO_2.5_/SrRuO_3_/SrTiO_3_ device having atomically uniform thickness and flat interfaces exhibited highly reproducible switching behavior with narrow distribution of the switching characteristics in both TE configurations (i.e. *Au-tip*(0.5 μm) and *Au*(20 μm), which was shown Fig. 2S (supplementary information). This atomically flat surface of the grown SrFeO_2.5_ surface drastically reduced the surface roughness effect on RS characteristics. These results also point out that the surface roughness can largely affect the RS device characteristics.

**Figure 6:**
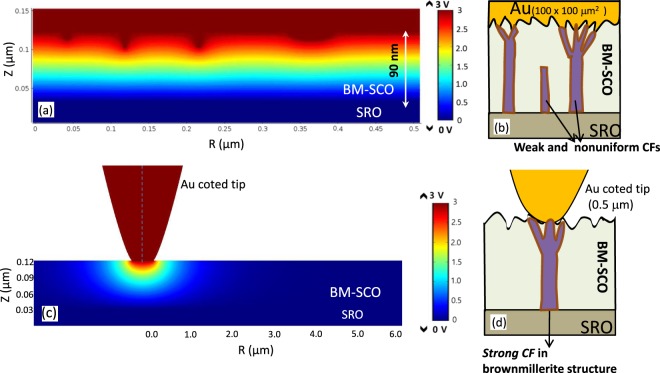
(**a**) A finite element simulation showing the electrical field distribution of the rough surface in the SrCoO_x_ thin film under a +3 V DC bias to the Au top electrode. (**b**) The rough interface between SrCoO_x_ and the top electrode can produce many local minimum paths for CFs having different lengths and strengths. (**c**) FEM calculated maps of the electrical field in the *Au-tip*(0.5 μm)/BM-SCO/SRO thin film. (**d**) The tip forces the exclusive generation of robust CFs just under the tip.

In general, redox-based RS is triggered by thermal or electric field-assisted oxygen anion migration resulting in a chemical change in the oxide layer, such as a valance state change, leading to RS^2,8^. However, the lack of control over oxygen ions in the CF leads to instability (cycle-to-cycle) in RS device performance. Nevertheless, the generation and distribution of oxygen ions can be significantly controlled by an applied local electric field^16,32^. Thus, to obtain knowledge of the field distribution in the rough surface of the BM-SCO thin film, a finite-element axis symmetry model was used to simulate the potential distribution in BM-SCO thin films, shown in Fig. 6(a). The model is a relatively simple representation of a physical system for potential distribution in the rough surface with randomly shaped asperities at the interface of the BM-SCO and TE. The height (2–6 nm) of the asperities was varied to match the BM-SCO film surface roughness height profile.

Because BM-SCO topography influences the TE-*Au*/BM-SCO interface, it will therefore affect the local potential distribution near the interface. This was illustrated in Fig. 6(a), which shows the potential distribution at an applied voltage of +3 V to the TE. We can observe a more concentrated electric field distribution in the vicinity of the asperities, especially at the sharp-edged type of asperity. These end points of the asperities can serve as a number of preferential points for generation of CFs from the SRO BE. Therefore, the rough surface of BM-SCO film can induce many local paths for the CFs, which leads to non-localized oxygen ion distribution over the whole device structure. As a result, the CFs may form randomly with some of the CFs having a poor oxygen ion concentration, as shown schematically in Fig. 6(b). This randomly generated multiple non-localized CFs in *Au*(100 μm)/BM-SCO/SRO device structures might be responsible for large fluctuation in the set curves, which is discussed in Fig. 5(a). Furthermore, It is well known that both the concentration of oxygen ions inside the CF and the width of the CF can significantly influence the retention property of a RS device. For example, based on a hopping percolation model, Takek *et al*. showed that high oxygen ion concentration in a CF region is the key factor for long data retention^28^. Subsequently, oxygen ions may be dispersed away from the weakly generated CFs over time, leading to retention fatigue (resistance decay to an intermediate state) of the *Au*(100 μm)/BM-SCO/SRO device.

On the other hand, the distribution of the electric field at the interface of the Au-coated tip/BM-SCO was visualized by a simple numerical calculation using a finite-element axis symmetry model, as shown in Fig. 6(c). The figure depicts the potential distribution between the tip and the SRO BE under a DC bias assuming an Ohmic contact between the Au tip and the BM-SCO layer. It can be observed that dense equipotential lines exist under the tip region of the BM-SCO thin film. In particular, a high electrical field concentration was observed near the edge of the Au-tip/BM-SCO area. The electric field concentration around the tip contact area would be even more intensified by applying a high DC bias voltage. Therefore, a well-confined electrical field at the tip edge region serves as a preferential starting point for metallic CFs from the BE through an electroforming process. Thus, those localized electrical fields around the *Au-tip*/BM-SCO interface under a positive tip bias will strongly attract the negatively charged oxygen ions from the BE-SRO. Therefore, the oxygen ion generation and distribution can be well controlled by a localized electrical field under the tip area, which leads to the generation of ‘*strong CF*s’, as shown in Fig. 6(d). The well-confined CFs with localized oxygen ion distribution lead to stable retention and high endurance with sharp set process in the *Au-tip*(0.5 μm)/BM-SCO/SRO device performance, which is shown Figs 4(b) and 5(b), respectively.

## Conclusions

In conclusion, by using a probe tip as a mobile top electrode, we verified the roughness effect of the interface on RS performance in a BM-SCO device. The probe tip-based devices show good RS properties including stable endurance and retention compared to devices based on a large electrode area. These behaviors are due to the confinement of oxygen ion distribution and the CF location by the localized electrical field distribution under the tip region. Finite-element simulation results further confirm a local confinement of the electrical field, which shows densely spaced equipotential lines below the probe tip regions. This simple point contact approach may provide further insight into understanding the underlying mechanism of resistive switching random access memory.

## Methods

### Resistive switching materials and device fabrication

For the device preparation, a 30~60 nm-thick epitaxial SRO BE was first deposited on an STO (001) substrate. The BE was deposited by pulsed laser deposition using a KrF excimer laser (repetition rate: 4 Hz; laser fluence: ~2.5 J/cm^2^) and at a substrate temperature of 750 °C. A ~90-nm-thick BM-SCO (001) thin film was then deposited on the SRO layer by pulsed laser deposition at a 600 °C substrate temperature, 10 mTorr oxygen partial pressure, approximately 2.0 J/cm^2^ laser fluence, and a 4 Hz repetition rate. E-beam evaporation was used to deposit a 2 mm × 5 mm-size Au pad which acted as the BE (it was connected to the bottom SRO layer) while 100 μm ×100 μm size Au squares were deposited, which acted as the TE for the devices with top electrode configuration. The details for the device fabrication can be found in our previous reports^25,26^.

### Structural and microstructural characterization

The epitaxial structure of the BM-SCO/SRO/STO (001) sample was investigated using high-resolution X-ray diffractometry (Bruker D8). The surface morphology of the BM-SCO thin films and STO (001) substrate was observed by atomic force microscopy (AFM) (XE-15, Park Systems). For TEM characterization, cross-sectional TEM specimens were prepared through focused ion beam (FIB) milling (Helios 650 FIB, FEI). The cross-sectional image of the device was acquired using TEM with an operating voltage of 200 kV (JEM-2100F, JEOL) equipped with an energy dispersive spectrometer (EDS).

### Measurement of the resistive switching characteristics

The current–voltage (*I-V*) characteristics were measured using a semiconductor device-parameter analyzer (Agilent B1540) under ambient conditions. During these measurements, bias was applied to the Au-coated probe tip, which acted as a mobile TE, while the BE was grounded.

### Simulations

A finite element method (FEM) was used to map the potential distribution in the device using the COMSOL Multiphysics 5.2 program. We adapted the numerical model approach from previously reported works on RS^18,33^.

## Supplementary information

Supplementary information
