# Supplementary material for: Confining vertical conducting filament for reliable resistive switching by using a Au-probe tip as the top electrode for epitaxial brownmillerite oxide memristive device
Source: Sci Rep. 2019 Feb 4;9:1188. doi: 10.1038/s41598-018-37986-6 (PMC6362224; doi:10.1038/s41598-018-37986-6)
Supplement: Supplementary file 1 — Supplementary information [file 41598_2018_37986_MOESM1_ESM.docx]

Supplementary information

Confining vertical conducting filament for reliable resistive switching by using a Au-probe tip as the top electrode for epitaxial brownmillerite oxide memristive device

Venkata Raveendra Nallagatla^a^, Janghyun Jo^b^, Susant Kumar Acharya^a^, Miyoung Kim^b^, and Chang Uk Jung ^a^*

*^a^ Department of Physics and Oxide Research Center, Hankuk University of Foreign Studies, Yongin 449-791, Korea*

*^b^ Department of Material Science and Engineering and Research Institute of Advanced Materials, Seoul National University, Seoul 151-747, Korea*

* Corresponding author: [cu-jung@hufs.ac.kr](mailto:cu-jung@hufs.ac.kr)

**Fig. S1.** (a) Surface morphology of the treated SrTiO_3_ (001) substrate with a clear step terrace. (d) AFM image of the as-grown SrFeO_2.5_ thin film surface with clear step terrace, the RMS roughness was ~ 0.45 nm.

**Fig. S2.** (a) *I–V* curves for the switching cycles the *Au-tip*(0.5 μm)/SrFeO_2.5_/SRO device. (b) Cumulative plots of the set and reset voltages for (a); (c) Cumulative probability graphs for the HRS and LRS for (a). (d) *I–V* curves for the switching cycles the *Au(20* μm)/SrFeO_2.5_/SRO device. (e) Cumulative plots of the set and reset voltages for (d); (f) Cumulative probability graphs for the HRS and LRS for (d). In contrast BM-SCO device, the atomically grown SrFeO_2.5_ memristive device exhibited high reproducibility and uniformity with both top electrode configurations (i.e. *Au-tip*(0.5 μm) and *Au*(20 μm ).
